# Strategies for Peripheral Nerve Repair

**DOI:** 10.1007/s43152-020-00002-z

**Published:** 2020-04-21

**Authors:** Matthew Wilcox, Holly Gregory, Rebecca Powell, Tom J. Quick, James B. Phillips

**Affiliations:** 1 Department of Pharmacology, UCL School of Pharmacy, University College London, 29-39 Brunswick Square, London, WC1N 1AX UK; 2 UCL Centre for Nerve Engineering, University College London, London, UK; 3 Peripheral Nerve Injury Research Unit, Royal National Orthopaedic Hospital, Stanmore, UK

**Keywords:** Nerve regeneration, Nerve biomechanics, Repair Schwann cells, Quantitative MRI, Quantitative neurophysiology

## Abstract

**Purpose of Review:**

This review focuses on biomechanical and cellular considerations required for development of biomaterials and engineered tissues suitable for implantation following PNI, as well as translational requirements relating to outcome measurements for testing success in patients.

**Recent Findings:**

Therapies that incorporate multiple aspects of the regenerative environment are likely to be key to improving therapies for nerve regeneration. This represents a complex challenge when considering the diversity of biological, chemical and mechanical factors involved. In addition, clinical outcome measures following peripheral nerve repair which are sensitive and responsive to changes in the tissue microenvironment following neural injury and regeneration are required.

**Summary:**

Effective new therapies for the treatment of PNI are likely to include engineered tissues and biomaterials able to evoke a tissue microenvironment that incorporates both biochemical and mechanical features supportive to regeneration. Translational development of these technologies towards clinical use in humans drives a concomitant need for improved clinical measures to quantify nerve regeneration.

## Introduction

Peripheral nerve injuries (PNI) are common following blunt or penetrating trauma, accounting for around 2% of all trauma cases [1, 2]. PNI are debilitating, leading to loss of sensation and movement and, in many cases, chronic pain for those affected, resulting in significant global socio-economic ramifications. The leading cause of PNI is vehicular collisions, and the people affected are predominantly young males [2].

Although the peripheral nervous system (PNS) has the capacity to regenerate to some extent, muscle function is often considered by patients to be incomplete [3]. Optimal functional reinnervation is dependent upon a sufficient number and quality of regenerating axons reaching their target within 1 year following injury [4–6]. Beyond this time period, functional outcomes are often disappointing [6]. This has been attributed to phenotypic changes in the microenvironment of the denervated nerve and muscle such that an incremental delay in reinnervation decreases the likelihood of functional recovery [4–6]. This is pertinent in proximal nerve injuries due to the slow rate of human nerve regeneration (approximately 1 mm/day) [7].

**Fig. 1:**
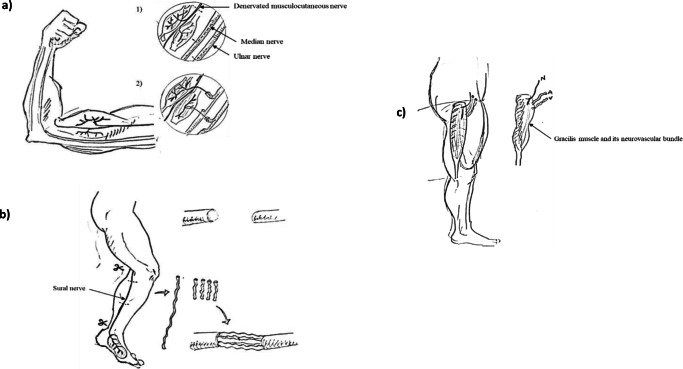
Reconstructive nerve procedures. **a** Nerve transfer is commonly deployed in severe proximal nerve injuries to restore elbow flexion (Oberlin’s nerve transfer). Synergistic donor motor nerves (fascicles ulnar and median nerves to wrist flexors) in close proximity to the injured nerve (musculocutaneous nerve) are dissected, divided and redirected to grow into the damaged nerve. **b** The nerve autograft is often selected to repair excessive acute gaps. A sensory (often sural) nerve is harvested and used to bridge the nerve gap. **c** Free functioning muscle transfer is elected in chronic nerve injuries. A donor muscle (such as the gracilis) and its neurovascular bundle are removed and grafted into the site of injury to restore function (such as elbow flexion). *N,* nerve; *A*, artery; *V*, vein

The most severe nerve injuries often benefit from operative intervention. Over recent decades, advancements in reconstructive surgery have improved functional outcomes. The nerve autograft, nerve transfer and free functioning muscle transfer (FFMT) (Fig. 1) are commonly deployed surgical strategies to restore function following severe PNI [4, 6, 8, 9]. These interventions aim to provide a tissue microenviroment that supports neural regeneration and/or minimise regeneration distances. Nerve autografting is associated with a number of limitations. Surgical transection of donor tissues leads to loss of donor nerve function, tissue remodelling and scar tissue formation. This permanently changes the biomechanical properties of tissues which can affect normal function of tendons, muscles and/or nerves [10]. Second, the availability of suitable donor sites where tissue for grafting can be liberated within patients is limited. Together, this has stimulated research into drug treatments that accelerate nerve regeneration and tissue engineered therapeutics which maintain the distal environment to support muscle reinnervation [11, 12].

**Fig. 2:**
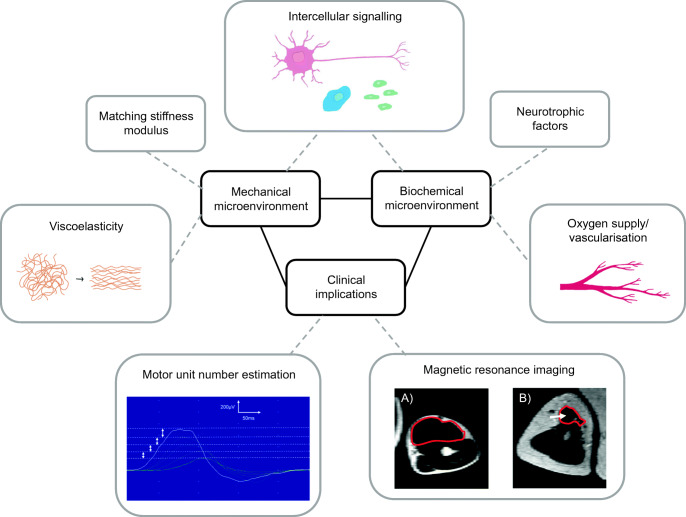
Strategies to improve peripheral nerve repair. Effective new therapies for peripheral nerve repair are likely to include engineered tissues and biomaterials that incorporate biological and mechanical features to support regeneration. Translational development of these technologies towards clinical use in humans requires improved clinical outcome measures of nerve regeneration. Motor unit number estimation: serial single motor unit potentials recorded from rat tibialis anterior using an incremental stimulation technique. Magnetic resonance imaging: T2-weighted MRI scans of uninjured and nerve injured biceps muscles from patient who sustained C5/6 Avulsion. **a** Uninjured biceps muscle (uninjured contralateral arm) outlined in red. **b** Subacutely denervated biceps muscle (3 months following injury) demonstrating increased signal (arrow) and atrophy of the biceps muscle (outlined in red) compared to **a**

To date, research to improve outcomes following peripheral nerve repair has largely focused upon either the biochemical or mechanical environment in isolation [13–16]. Therapies that incorporate multiple aspects of the regenerative environment are likely to be key to improving therapies for nerve regeneration (Fig. 2). However, this represents a complex challenge when considering the diversity of biological, chemical and mechanical factors involved. Mathematical and/or in silico modelling can be utilised to resolve this complexity in order to inform clinicians and researchers about how to optimise treatments. In addition, clinical outcome measures following peripheral nerve repair which are sensitive and responsive to changes in the tissue microenvironment following neural injury and regeneration are awaited. Addressing these challenges will important in developing effective therapies for the treatment of PNI. This review will consider the key aspects of the nerve tissue microenvironment that underpin development of new strategies for peripheral nerve repair. In particular it will cover biomechanical and cellular considerations required for development of biomaterials and engineered tissues suitable for implantation following PNI, as well as translational requirements relating to outcome measures for testing success in patients (Fig. 2).

### Mimicking the Biomechanical Properties of the PNS

The biomechanical properties of peripheral nerves are vital to the development of better surgical options and biomaterials for nerve repair constructs. Peripheral nerve structure is arranged systematically, in which axons supported by Schwann cells and endoneurial tissue are bound together in fascicles by perineurium, a layer composed of concentric flat perineurial cells containing tight junctions [17]. A number of fascicles, in conjunction with blood vessels, are grouped together and encompassed by epineurium to form a nerve. Collagen fibrils are arranged longitudinally within the endoneurium and throughout the perineurium and epineurium, where they form a meshwork of larger fibre structures that provides strength and flexibility [18]. This structure allows the nerve to function effectively under the stresses imparted by normal movement, bestowing tensile strength and elasticity [19–22]. During movement, peripheral nerves glide relative to the surrounding muscle and bone, and nerve fascicles slide independently of one another [23, 24]. When the whole nerve is under tensile load, it both elongates axially and compresses across the circumference non-linearly – the tissue elongates faster and the circumference decreases more quickly at lower tensile loading [25].

Human nerve tissue is relatively soft – the modulus of ulnar nerve has been measured as 12 kPa in vitro and 54 kPa in vivo [26]. Like the majority of biological tissue nerve tissue displays viscoelastic behaviour, which means they can be considered to have both elastic components and viscous components [27]. Elasticity is evident in human nerve, which can be elongated up to 6% without damage [28], and in fact nerves are under constant physiological strain. In situ measurements of rabbit tibial nerve demonstrate minimal stress at a strain of 11% [21], and rat sciatic nerve retracts around 11% when severed [29]. However, the window of tolerated force is narrow. Kwan and co-workers found that increasing the percentage strain on rabbit tibial nerve in situ from 6 to 12% increased the likelihood of non-recoverable conduction block, and increasing applied stress up to 1.75 MPa reduced the compound nerve action potential to less than 20% of the baseline value after a 1-hour recovery period [21].

Nerve tissue also demonstrates a property common to viscoelastic materials in that the speed of elongation determines the ability of the tissue to withstand strain. Ikeda et al. stretched the rabbit sciatic nerve by 30 mm through femur elongation at 0.8 mm/day, which was found to cause little nerve damage, whereas 2.0 mm/day tended to cause recoverable damage, and 4.0 mm/day tended to produce irreversible damage [30]. Other viscoelastic behaviours exhibited by peripheral nerves include stress relaxation, in which the stress needed to maintain a certain strain reduces with time; and creep, where the strain produced by a set stress will increase with time. These behaviours have been demonstrated in cadaveric human sciatic nerve [31], and are valuable in allowing nerve tissue to adapt to stress from body movement.

It is important to note that injury may alter the mechanical behaviour of nerves. Mouse sciatic nerve after crush injury demonstrated increased nerve strength and stiffness and decreased elasticity compared to uninjured nerves, effects which increased up to 12 days post-crush for strength and stiffness and 24 days post-injury for elasticity [32]. In cadaveric human digital nerve, crush injury had no effect on the ultimate tensile strength, stiffness, maximum stress or strain of the tissue [33]. However, this result is unsurprising given that post-injury increase in stiffness and loss of elasticity may be due to fibrosis in the neural tissue [24, 34], a physiological reaction which would not be present in cadaveric samples.

An understanding of native nerve mechanics is important if tissue engineers are going to replicate it effectively in biomaterial constructs, used as an alternative to the autograft to bridge long gaps in nerve tissue. As is evident in this review and others [24], much of the recent mechanical data available are from animal models and relatively few studies use human tissue. The mechanical properties of native nerve must be more thoroughly investigated and reproduced as closely as possible in the design of biomimetic constructs for nerve repair.

To imitate the complex mechanical environment of peripheral nerve, the material should be relatively soft to match the modulus of nerve tissue and elastic to accommodate the considerable strain that peripheral nerve endures without damage. The construct material must also maintain enough stiffness to prevent the surrounding tissue from swelling into the gap between nerve ends and blocking the path of regenerating axons. This balance is difficult to strike – the results from clinical trials of three FDA-approved bioabsorbable peripheral nerve conduits have been published in peer-reviewed journals [35], and even these constructs may not possess appropriate mechanical properties. An independent study comparing them with an autograft in a 10-mm rat sciatic nerve gap found the polyglycolic acid conduit to have collapsed completely after 12 weeks in all animals [36]. The authors note that this could be attributed to a size mismatch in using conduits intended for human nerves in a rat model, however also point out that these conduits were used successfully in human facial nerve [37] which would also have a relatively small diameter compared to the conduit. Constructs which are too stiff can be equally detrimental to recovery. Matching the mechanical modulus of a rat peripheral nerve implant environment by coating poly(dimethylsiloxane) (PDMS) implants in soft (< 10 kPa) polyacrylamide or PDMS gel was found to suppress inflammation and reduce foreign body response compared to implants with stiffer moduli [38]. In general the mechanical microenvironment is hugely impactful on the cellular environment – the lineage and phenotype of mesenchymal stem cells (MSCs) has been shown to be specified by matrix elasticity, and softer matrices found to encourage a neurogenic phenotype [39].

Belanger and co-workers recently designed a trilayered electrospun silk fibre material for nerve repair which utilised aligned outer layers for axonal guidance and a randomly orientated inner layer [15]. The trilayer material had comparable stiffness to a purely aligned material (and to rat sciatic nerve) and demonstrated improved ductility, which the authors suggest and is explained by the rearrangement and alignment of the randomly orientated fibres in the direction of tensile stress. The authors also suggest that the trilayer material demonstrated better surgical handling properties due to increased tear strength [15]. Electrospun fibre conduits with and without alignment have also been developed using polycaprolactone and chitosan [40]. The researchers found the fully aligned conduit had improved compression properties but reduced tensile strength due to sudden breakage of the orientated fibres. In a 10 mm rat sciatic nerve model, the aligned conduit displayed a number of improved in vivo regenerative indicators compared with the randomly aligned material [40].

A number of technologies based on synthetic materials have been developed to more closely imitate the mechanical behaviour of biological tissues. Implantable electric devices which record and modulate signals in the PNS, known as peripheral nerve interfaces, are currently designed using flexible and stretchable silicone-based elastomers such as PDMS, which provide high extensibility and relatively low Young’s modulus values [41]. A system which can reproduce strain stiffening behaviour has been developed based on brush- and comb-like polymer networks and allows precise replication of specific tissue characteristics based on network strand length, polymer grafting density and side chain length [42]. Similarly, a combination of polyethylene glycol and branched polyethylenimine has been used to create a strain stiffening and self-healing flexible hydrogel which mimics the mechanical response of a biological system to stress [43]. Although these systems and others may have application in materials for nerve constructs, mechanical performance to match that of the nerve microenvironment is in general rarely considered during material development in constructs for nerve repair. However, current work in mathematical modelling is aiding our understanding of the complex mechanical environment of neural tissue and is becoming a vital tool in development of biomaterial constructs. For example, Giannessi et al. used a polynomial strain energy function to model the mechanical response to stretch of nerve from different species and built in silico models of porcine nerve and *Aplysia* cerebro-abdominal tissue [44]. The authors note that although the model was focused on nerve hyperelasticity, elements such as viscosity could be included to allow computational modelling of nerves during regeneration through scaffolds.

### Creating a Tissue Microenvironment that Supports Regeneration

The cellular components of a peripheral nerve have distinct and essential roles to play during peripheral nerve injury and repair, the key component being Schwann cells. Only hours after an axonal transection injury, Schwann cells transform to a ‘repair’ phenotype. These do not produce myelin and undergo autophagy to break down existing myelin. Expression of growth factors, such as glial cell line-derived neurotrophic factor (GDNF) and nerve growth factor (NGF), is upregulated, as well as cytokines that can recruit macrophages. Repair Schwann cells adopt a longer bipolar morphology as they proliferate [45••], and form tracks called bands of Büngner which guide new axons and prevent misdirection of reinnervation [46]. Wallerian degeneration, where axons and myelin degrade within the distal nerve, starts after an injury. Macrophages infiltrate at this stage to phagocytose cellular and tissue debris, establishing a pro-regenerative environment for new axon growth. In larger nerve gaps, an autograft is the current clinical standard of care for reconstruction [47]. Inserting a length of healthy nerve as an autograft initiates the repair process in the grafted nerve bridge, transferring a column of repair Schwann cells into the injury site that can guide axon growth from the proximal stump. Mimicking this biological microenvironment provides the motivation for peripheral nerve repair through tissue engineering.

There are two main roles of repair Schwann cells in the peripheral nerve injury microenvironment – nerve regeneration support through the release of neurotrophic factors and physical guidance of the regenerating axon. Cell therapies so far have focused a great deal on neurotrophic factor release. Mesenchymal stem cells (MSCs) have been widely used in research and are an attractive option due to availability, ability to differentiate into neural cell types and the expression of neurogenic and immunoprotective factors. However, MSCs are also ill-defined and are often a mixed population from a variety of sources – from the most common bone marrow [48] and adipose-derived [49, 50] to Wharton’s jelly [51] and tonsil-derived [52]. High variability in numbers, function and sources makes large-scale expansion more complex. More defined cell sources are embryonic stem cells (ESCs) and induced pluripotent stem cells (iPSCs). As iPSCs are created from adult cells [53, 54], they are advantageous over embryonic stem cells (ESCs) due to reduced ethical complications which would present difficulties when implementing an ESC-based therapy worldwide [55]. iPSC therapy has been pushed forward to clinical trials in Japan [56•], and the US [57], and so far this type of cell therapy has been reported as safe [58].

iPSCs can be differentiated into Schwann cells via a precursor stage [59, 60], with Schwann cells being seen as the ideal cell type for therapy due to the key role they have in the repair process [45••, 46, 61]. The repair Schwann cells in the nerve autograft show greater similarity to ES cells than neural crest cells [62]. For this reason, it is possible that cells at earlier stages of differentiation will support regeneration to a greater degree than terminally differentiated Schwann cells [60]. iPSC-derived Schwann cell precursors and Schwann cells have shown significant functional improvement compared to controls without cell therapy [59], with key outcomes being neurotrophic factor release and increased myelin formation.

Extracellular vesicles, exosomes and secretomes have benefits over cell therapies because of the challenges around patient matching for allogeneic cell therapies, as well as manufacturing challenges regarding cell supply and quantity. In one study, a 10 mm gap in rats was repaired with a chitin conduit alongside injection of exosomes from gingiva-derived mesenchymal stem cells. The group treated with exosomes showed equivalent recovery in nerve fibre myelination and muscle weight to the autograft group after 12 weeks, which was significant compared to the empty conduit [63]. Exosomes isolated from differentiated ADSCs have been shown in vitro to reduce apoptosis of Schwann cells [64] and those from undifferentiated ADSCs promote neurite outgrowth of NG108-15 cells [65]. This suggests that a key role of transplanted stem cells in peripheral nerve repair is the release of neurotrophic factors; although without living cells present to release neurotrophic factors continuously, secretome-based therapies might be limited to short-term effects.

Neurotrophic factors, although essential to the regeneration of an axon, must be delivered in a controlled manner. For example, excess levels of GDNF can be detrimental to nerve repair, causing nerve sprouting and axon entrapment [66]. Neurotrophic factors have potential to be delivered as a drug, although gene edited cell therapies that allow controlled release of neurotrophic factors are an attractive option. By combining expression of specific neurotrophic factors such as GDNF [66] or the upstream transcription factor c-Jun [67•] with a Tet-On/Tet-Off system, the delivery can be carefully controlled to avoid overexpression and off-target reinnervation [66].

Neurotrophic factor release is not the only role of repair Schwann cells. Repair Schwann cells also interact with other cells at the injury site to ensure successful reinnervation. Dun et al. have found complementary attraction and repulsion signalling interactions between repair Schwann cells, macrophages and fibroblasts via the Slit-Robo pathway [68, 69], which are essential for formation of the nerve bridge and directing regenerating axons. Macrophages surrounding the nerve bridge express Slit3, which binds to the Robo1 expressed by repair Schwann cells and acts as a repellent force ensuring repair Schwann cell migration remains directed along the nerve bridge. Repair Schwann cells also interact with blood vessels, which form early across the nerve bridge and help guide Schwann cells [70]. Blood vessel formation is also regulated by macrophages releasing VEGF [70], as well as the interaction between Robo1 on blood vessels and Slit3 on macrophages [68]. There is a network of cell-cell interactions in a nerve bridge that together result in successful regeneration. Mimicking these intercellular interactions will be an important part of ensuring new engineered tissue therapies succeed.

Nerve graft hypoxia is not well characterized. Injury results in the damage of blood vessels, leading to hypoxia around the peripheral nerve injury bridge. Short-term hypoxia may induce some advantageous changes – it has been found to enhance c-Myc transcription in cell lines [71], and to promote vascularisation [72–74], but long-term hypoxia will lead to cell death [75]. VEGF, expressed by macrophages in the nerve bridge [70], is essential to promote blood vessel growth [76]. Indeed, just delivering VEGF can improve functional outcomes following nerve injury in mice [77], and having therapeutic cells that promote angiogenesis in a construct are likely to improve survival of the implanted cells and be beneficial for nerve repair [73].

The biological microenvironment in a peripheral nerve injury site involves a complex network of cells that support axon regeneration through neurotrophic factor release and physical guidance cues. The interaction between cells in the nerve repair site and those in the surrounding tissue are essential to successful regeneration, ensuring both axon regeneration and vascularisation are supported and guided. Mimicking this microenvironment will involve a combination of existing technologies, and mathematical modelling can untangle the complexity to ensure the most important factors that can be prioritised [78].

### Challenge of Clinical Translation

A number of therapeutics for the treatment of PNI have been developed in animal models, including engineered tissues that mimic the regenerative microenvironment found in the nerve autograft and distal nerve segment [73, 79–82]. However, there are many challenges associated with the clinical translation of these and other therapies with the potential to improve nerve repair outcomes in patients. First, little is known about the in vivo biology of human nerve regeneration. Second, assessments that are sensitive and responsive to sub-clinical changes in the tissue microenvironment are still under development.

#### Human Nerve Regeneration

Whilst a great deal is known about the cellular and molecular signals that underpin nerve regeneration in rodents [45••, 83], it is unknown whether these are mimicked in humans. There are a number of challenges associated with studying human nerve regeneration which are not encountered in animal models. It is challenging to liberate human nerve samples for study in the laboratory without creating significant patient morbidity. Even when there are opportunities to retrieve finite amounts of excess human nerve from some reconstructive nerve procedures (such as the nerve autograft and nerve transfer) for experimental use, there are a number of perioperative variables that must be considered. A recent study demonstrated the deleterious effect of surgical antiseptics and time delays (as short as 3 min) on the quality and quantity of RNA isolated from human nerve samples [84]. Additional advances in the techniques used to study the nerve tissue microenvironment in humans are required in order to understand the differences and similarities between rodent models and their human patient counterparts. Since human nerve tissue is likely to remain a rare resource for experimental study, approaches that maximise the yield of RNA and the detection of other tissue biomarkers will be valuable, as will new clinical assessment measures and non-invasive imaging.

#### Clinical Assessments

Clinical assessments of nerve injury are ultimately an assessment of function, i.e. the extent to which damaged neurons have successfully reinnervated their target organs to restore sensation and/or control of muscle contraction. The recovery of motor function is universally assessed using manual muscle testing and in particular the Medical Research Council (MRC) grading system (Table 1) of peak volitional force. This assessment of muscular function has been shown to be limited for a number of reasons with over 96% of recordings being classified as MRC Grade 4 [86]. This has stimulated a shift towards the use of continuous measurements of peak volitional force using handheld dynamometry [87]. However, patient reported experiences of muscle reinnervation have demonstrated that an earlier onset of fatigue is a central theme of muscle reinnervation [88–90]. In the context of motor function, muscle fatigue can be defined as the inability to sustain force over time [91]. Recent studies have shown that surface electromyography (EMG) measurements during sustained and repeated isometric contraction of reinnervated muscle may be used to monitor muscle fatigue [88, 90]. Adoption of these metrics into clinical assessments of muscular function will be the key to driving advancements in motor recovery therapy. Diagnostic tests such as neurophysiology and imaging may also present useful tools to quantify changes in the tissue microenvironment associated with nerve regeneration.

**Table 1:** The MRC grading system of muscle power [85]

MRC Grade	Clinical presentation
0	No movement
1	Flicker of movement
2	Active movement when gravity removed
3	Active movement against gravity only
4	Active movement against resistance
5	Normal muscle power

#### Neurophysiology

Nerve conduction studies (NCS) and EMG are the first-line tests used by clinicians to determine the location and extent of nerve damage [92]. NCS measure the speed and amplitude of currents passed along nerves, whilst EMG provides an impression of nerve function and its interaction with the muscle [93]. However, many of these neurophysiological changes provide limited information about the functional microenvironment at the interface between regenerated nerve and muscle, i.e. the number of functional motor units. Motor unit number estimation (MUNE) is a neurophysiological test that estimates the number of motor units (MUs) innervating a muscle. MUNE is based on the phenomenon that it is possible to recruit individual MUs by incrementally increasing stimulation to the nerve and its muscle [94] (Fig. 2). MUNE has been utilised as a tool to characterise the dynamics of a number of pathologies associated with muscle denervation such as amyotrophic lateral sclerosis (ALS) and spinal muscle atrophy (SMA), and has been used as a primary outcome measure in clinical trials [95–97]. Application of MUNE in muscle reinnervation is not well documented, but a number of methods to determine MUNE have been reported [95, 98, 99]. If changes in the tissue microenvironment during nerve regeneration can be correlated with MUNE, this might provide a useful minimally invasive way to quantify both animal and human nerve regeneration [100••].

#### Imaging

Imaging is widely employed in the clinical work-up of patients with central nervous system (CNS) pathologies but is not in widespread use for peripheral nerve disorders. This is despite a number of studies demonstrating changes evident on MRI scans that are associated with the injured nerve and skeletal muscle [101]. It is hoped that these markers may provide sensitive and responsive measures of changes in the tissue microenvironment associated with injury and regeneration.

#### MRI of Peripheral Nerve Lesions

Uninjured nerves demonstrate a signal that is isointense or moderately hyperintense compared to the surrounding muscle on a T2-weighted (T2-w) image. By extension, it is often difficult to distinguish between an uninjured nerve and its surrounding muscle. However, when an injury has caused axonal loss within the nerve trunk, it is possible to distinguish between the nerve and surrounding muscle. The injured nerve will demonstrate an increase in T2 relaxation time and will appear “bright” on a T2-w scan as soon as 24 h following injury [102, 103]. The signal change regresses back towards normal levels following successful nerve regeneration and is well correlated with the return of motor function [104, 105]. In addition, these changes on MRI precede EMG markers associated with recovering voluntary activity [105, 106]. It remains largely unknown what these changes in T2 relaxation time correlate to within the tissue microenvironment although an increase in extracellular volume is thought to be responsible [104, 105, 107].

Diffusion tensor imaging and tractography provide a graphical representation of the microanatomy of nerves [108]. However, the sensitivity of these techniques must be improved in order to image changes in the nerves associated with injury or compression following trauma [108]. It must also be elucidated what relationship these images have with function.

#### MRI of Denervated Muscle

Normal muscle appears as an intermediate signal on T1-weighted (T1-w) and T2-w images. Denervated muscle demonstrates hyperintense signals on fluid sensitive MRI sequences (such as short tau inversion recovery (STIR) and turbo inversion recovery magnitude (TIRM)) [103, 109–111]. Upon successful nerve regeneration, the hyperintense signal regresses back towards normal levels [101, 106, 112], and these changes precede EMG markers of recovery [101, 106, 112]. The cellular and molecular mechanisms in the tissue microenvironment responsible for these changes remain poorly understood, and quantification in a standardised model of muscle reinnervation is required.

A number of changes in the tissue microenvironment take place following muscle denervation. The loss of neural trophic support leads to muscle atrophy and fat infiltration (Fig. 2). In prolonged denervation, this ultimately leads to a loss of muscle mass. The recovery of rat gastrocnemius muscles was found to range from 19 to 100% of the uninjured contralateral side following immediate nerve repair [113–115]. However, the outcome is much poorer when nerve repair is delayed beyond 3 months following injury, with muscle wet weight recovering to only 10–20% of the uninjured contralateral side [113–115]. Changes in muscle wet weight and MRI signal could theoretically be used to monitor and predict functional recovery following peripheral nerve repair. However, measurement of muscle wet weight in humans is not possible, although recent pilot studies have explored muscle volumetric changes associated with facial muscle reinnervation [116, 117].

It would be useful to quantify changes in MRI signal and muscle volume in a model of muscle reinnervation. Understanding the relationship between these MRI changes and objective and subjective measurements of muscular function will help with the validation and widespread adoption of MRI as a clinical and research tool in PNI.

#### Ultrasound

Ultrasound (US) can be used in the acute phase of nerve injuries where it has a role in identifying the level of injury and entrapment pathologies [118]. High-resolution US has been shown to be a highly sensitive method for differentiating between axonotmesis and neurotmesis injuries preoperatively [110, 112]. However, this technique is highly operator-dependent, and imaging of deeper nerves is often challenging.

## Conclusions

Effective new therapies for the treatment of PNI are likely to include engineered tissues and biomaterials able to evoke a tissue microenvironment that incorporates both biological and mechanical features supportive to regeneration (Fig. 2). Translational development of these technologies towards clinical use in humans requires improved understanding of the human nerve and muscle microenvironment and drives a concomitant need for improved clinical measures to quantify nerve regeneration. This will require the engagement and collaboration of multidisciplinary teams that incorporate scientists, engineers, clinicians and mathematical modellers in order to drive innovation and improve therapeutic options in this field.
